# Submucosal Lipomas Causing Intussusception and Small Bowel Obstruction: A Case Report

**DOI:** 10.7759/cureus.3692

**Published:** 2018-12-05

**Authors:** Mohamed Ahmed, Saba Habis, Rasha Saeed, Ahmed Mahmoud, David Plurad

**Affiliations:** 1 Surgery, Riverside Community Hospital, Riverside, USA; 2 Internal Medicine, Riverside Community Hospital, Riverside, USA

**Keywords:** small bowel obstruction, submucosal lipoma, intussusception

## Abstract

Intussusception is a rare cause of bowel obstruction caused by both benign and malignant pathology. We are presenting the case of an elderly patient who had been diagnosed with irritable bowel syndrome for many years prior to presenting to us with ileocecal intussusception causing a small bowel obstruction. Laparoscopic resection of the terminal ileum and cecum was performed. Pathology revealed multiple submucosal lipomas as the underlying cause.

## Introduction

Intussusception is defined as the invagination of a proximal bowel segment into the lumen of an adjacent distal segment. It is the cause of 1% - 5% of small bowel obstructions in adults [1]. The incidence is two to three cases per 1,000,000 per annum. The first reported case was made in 1674 by Dr. Paul Barbette of Amsterdam. The age of presentation is highly variable, ranging from the neonatal period to the seventh decade of life [2]. Intussusception in the ileocolic region is most commonly presented in children and is uncommon in adults [3]. Most patients with intestinal lipomatosis are asymptomatic; however, some present with subacute intermittent obstruction, colonic perforation, and rarely, intussusception [4]. The lead points for the intussusceptions are attributable to benign, malignant, or idiopathic causes [5].

## Case presentation

**Figure 1:**
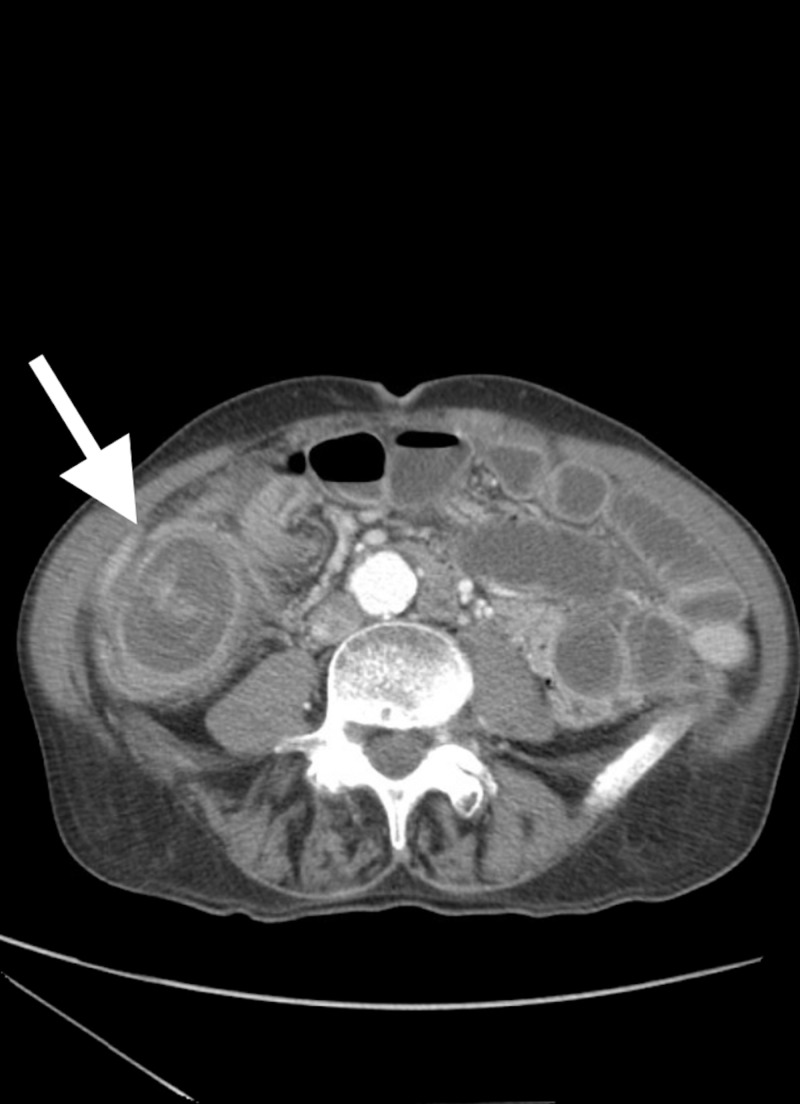
Axial computed tomography (CT) scan showing intussusception

A 67-year-old Caucasian male, who been diagnosed for many years with irritable bowel syndrome, presented to our emergency room with a five-day history of abdominal pain, nausea, vomiting, and obstipation. The patient's past medical history also included hypertension and an appendectomy. His examination revealed a soft, distended abdomen. A computed tomography (CT) scan of the abdomen and pelvis was consistent with small bowel obstruction secondary to ileocecal intussusception (Figure 1).

**Figure 2:**
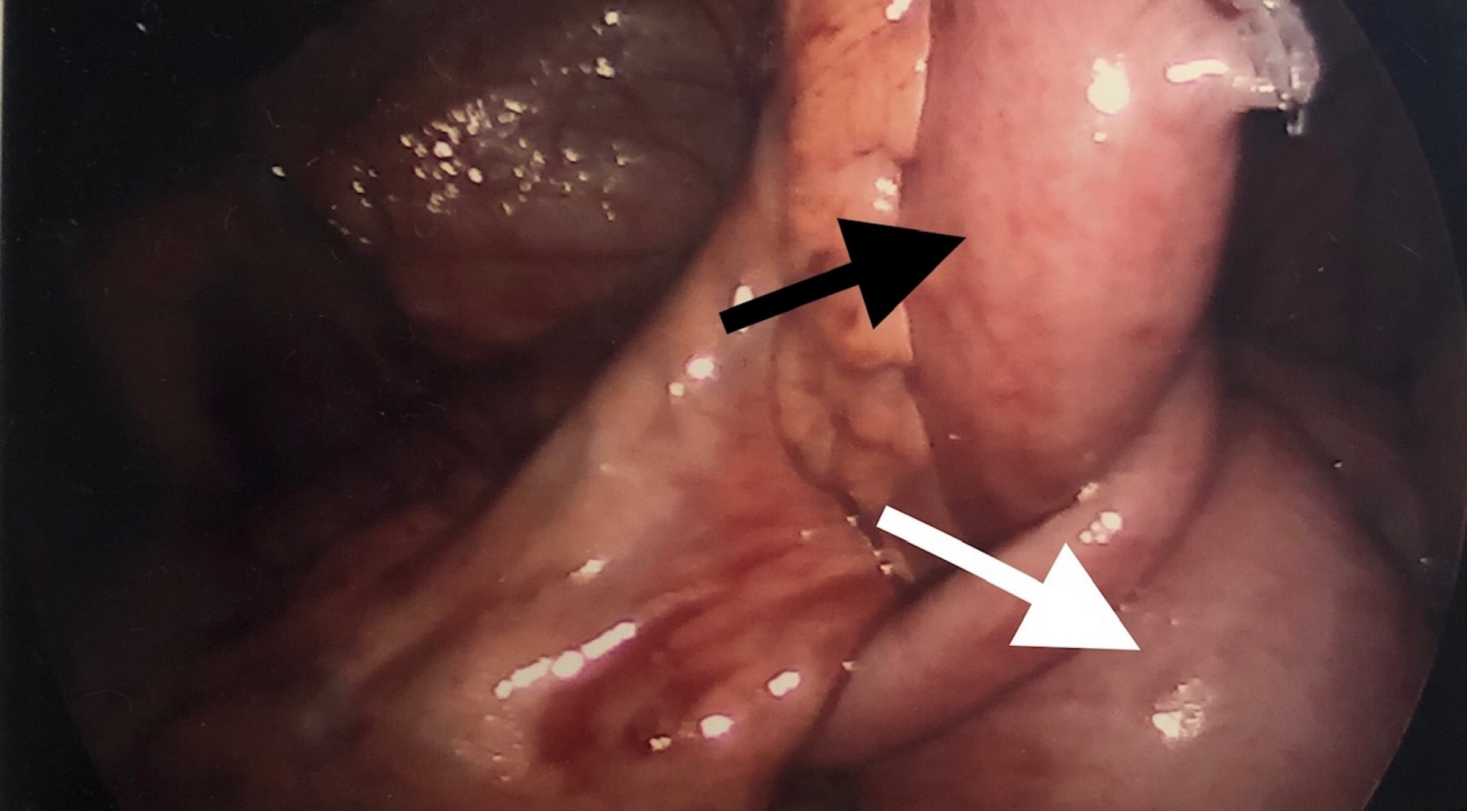
Laparoscopic view of the ileocecal intussusception The terminal ileum (black arrow); the cecum (white arrow)

He was admitted to the hospital, and a laparoscopic right hemicolectomy with resection of the terminal ileum and an extracorporeal side-to-side stapled ileocolic anastomosis was performed (Figure 2). The patient did well and was discharged from the hospital.

**Figure 3:**
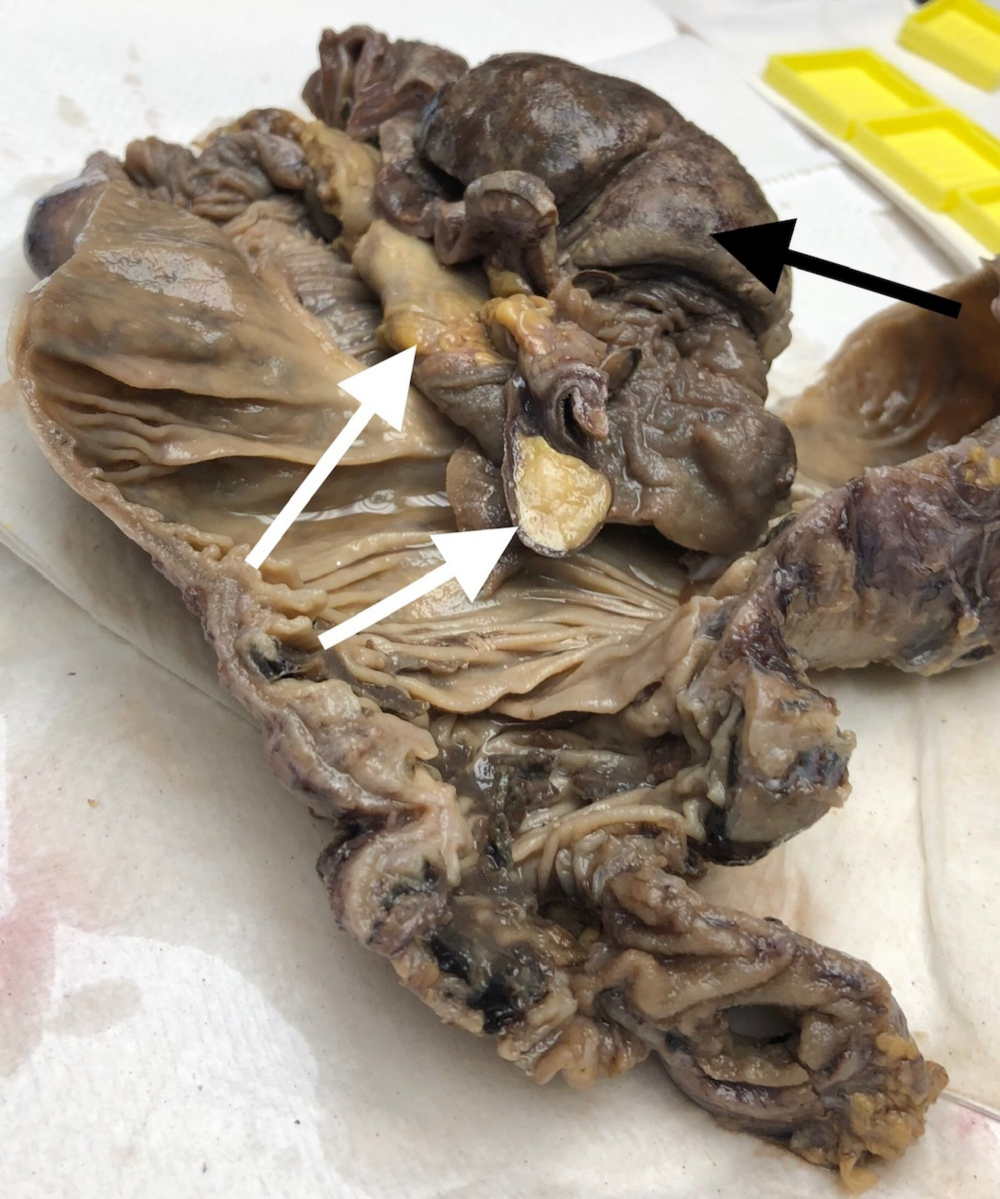
Gross pathology revealed two submucosal lipomas (white arrows) Ischemic terminal ileum loop (black arrow)

**Figure 4:**
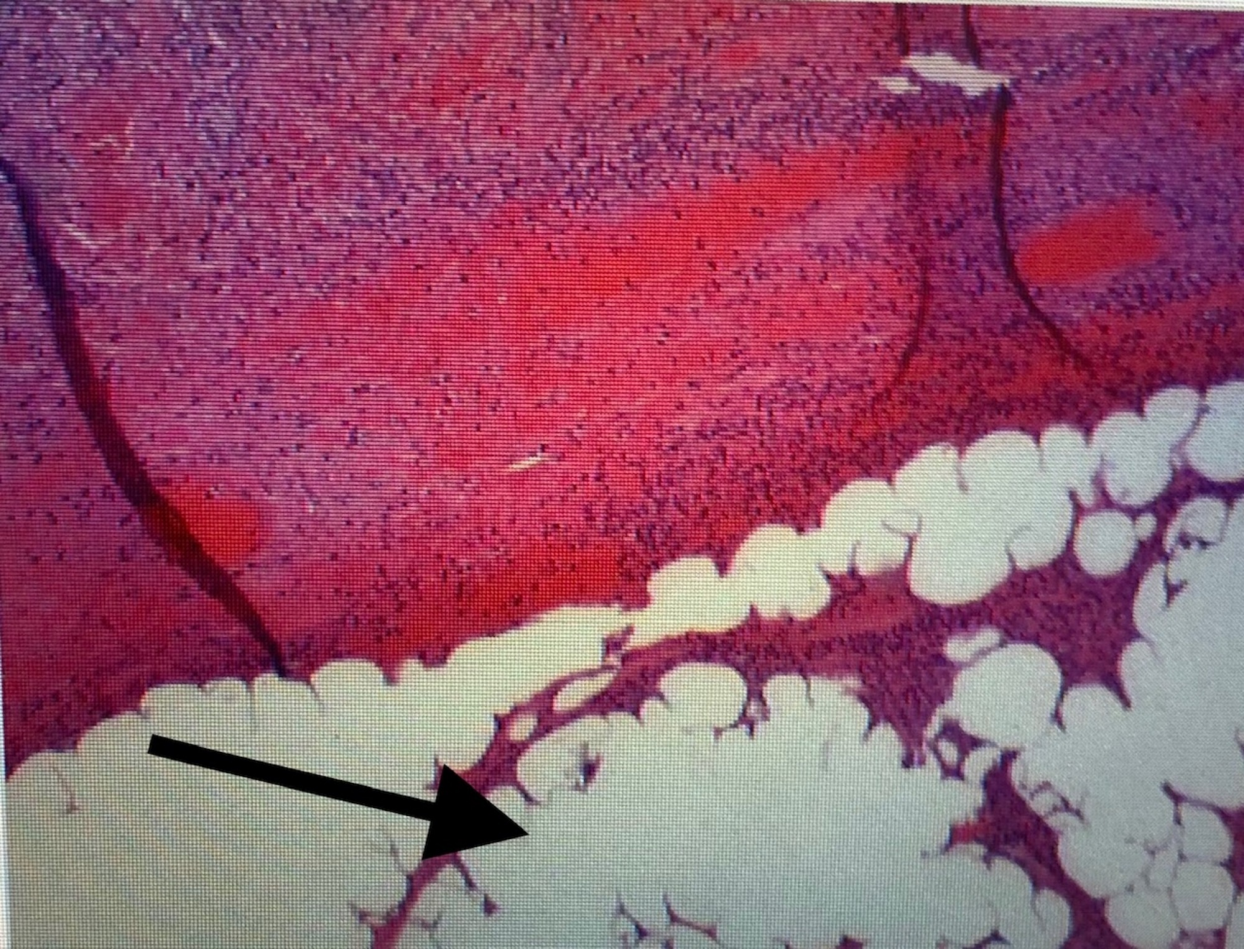
Histopathology revealed mature adipose tissue consistent with a lipoma (black arrow)

Gross pathology revealed two submucosal lipomas with an ischemic terminal ileum loop (Figure 3). Histopathological examination of the leading point indicated mature adipose tissue consistent with a lipoma (Figure 4).

## Discussion

The incidence of intussusception is two to three cases per 1,000,000 per annum. Intussusception is defined as the invagination of a proximal bowel segment into the lumen of an adjacent distal segment and being the cause of 1% - 5% of small bowel obstructions in adults [6]. Intussusception in the ileocolic region most commonly presents in children and is uncommon in adults [3]. The lead points for the intussusceptions are attributable to benign, malignant, or idiopathic causes [6]. Most patients with intestinal lipomatosis are asymptomatic; however, some present with subacute intermittent obstruction, colonic perforation, and rarely, intussusception [7]. Repeated intussusceptions can happen in a patient with multiple submucosal lipomas [8]. A CT scan of the abdomen and pelvis has a 100% specificity and 87% sensitivity in adults [9]. While colo-colonic intussusception is predominately caused by malignancy (70% of cases), 70% of small intestine intussusceptions are caused by a benign pathology. Primary resection, open or laparoscopic, is the treatment of choice, especially in patients over 60 years old, because of the high incidence of malignancy [10].

## Conclusions

Intussusception in adults is rare and is usually caused by an underlying tumor, most often malignant. Reduction in adults of an ischemic bowel and possible malignancy should not be attempted. Surgical resection either via laparoscopy or laparotomy is the best therapeutic option. A thorough gastrointestinal evaluation is also recommended prior to assigning the diagnoses of irritable bowel syndrome. In our case, the cause was multiple submucosal lipomas. 
